# A multiple regression based method for indirect compensation of hemispherical resonator gyro temperature error

**DOI:** 10.1038/s41598-023-31868-2

**Published:** 2023-04-13

**Authors:** Li Xin-san, Li Can, Shen Qiang, Wang Li-xin, Li Shuan-zhu

**Affiliations:** 1 Rocket Force University of Engineering, Xi’an, 710025 China; 2 Shandong Huali Electromechanical Co., Ltd, Wenshang, 272500 Shandong China

**Keywords:** Aerospace engineering, Electrical and electronic engineering

## Abstract

In order to improve the measuring accuracy of the Hemispherical Resonator Gyro under variable temperature, aiming at the problem of "external temperature is unavailable and internal temperature is unmeasurable," a multiple regression based method is proposed for compensating temperature error in the gyro. The relationship between the internal temperature and the resonant frequency of the gyro is analyzed theoretically. According to a constant temperature experiment, a linear relationship between them is derived based on the least square method. The analysis of a temperature-rising experiment shows that the correlation of the gyro output with the internal temperature is much higher than that with the external temperature. Therefore, taking the resonant frequency as an independent variable, a multiple regression model is established for compensating the temperature error. The compensation effect of the model is verified by temperature-rising and temperature-dropping experiments, which show that the output sequence before compensation is not stable, while it is stable after compensation. After compensation, the drift of the gyro decreases by 62.76% and 48.48%, respectively, and its measuring accuracy becomes equivalent to that at the constant temperature. The experimental results verify the feasibility and effectiveness of the model developed for indirect compensation of temperature error.

## Introduction

Compared with the traditional rotor gyroscope, the hemispherical resonant gyroscope has the advantages of long service life, high reliability, and good long-term stability^1^. So far, it has been successfully applied to a certain type of aerospace vehicle. With the maturity and development of technology, the hemispherical resonant gyro will have a broader application prospect in aviation, aerospace, and missile weapons. The resonator of the hemispherical resonator gyro is made of a quartz material. Since the physical properties of quartz materials are easily changed with temperature, the measuring accuracy of the hemispherical resonator gyro also changes with temperature^2,3^. The research on compensating temperature error in the hemispherical resonator gyro is of great significance for improving its measuring accuracy under variable temperature conditions.

In engineering applications, there are generally two ways to suppress the temperature drift in gyroscopes: passive suppression of temperature drift by hardware and active compensation of temperature error by software^4^. The passive suppression method by hardware is a temperature control method to eliminate any temperature change, so as to maintain a constant temperature field in the working environment of the gyro^5^. The advantage of this method is that it can eliminate the drift caused by temperature change, thus suppresses the sources of errors in the control circuit of the gyro and in other temperature sensitive areas. The disadvantages of the method are that the cost of hardware materials required for heat shield is high, and a large space is required for the hardware. In contrast, the active compensation by software has the advantages of saving equipment space, no additional material cost, and fast application effect. So, at present it is the most concerned method for compensating temperature error. In reference^6^, an AR multivariable model for temperature error in the hemispherical resonant gyro was established with the ambient temperature as the variable for off-line realization of the compensation of temperature error in the gyro. However, this method takes the ambient temperature as the variable, which cannot reflect the actual temperature inside the gyro. Besides, The temperature compensation model only takes the temperature difference as the independent variable, without considering the temperature, temperature gradient and coupling terms, which is difficult to fully reflect the temperature change rule. In reference^7^, influence of temperature on the resonant frequency was analyzed, and a self-compensation method for temperature drift of zero bias was established. After compensation, the zero bias drift was reduced from 30 to 2.8°/h. This method can effectively reduce the temperature drift in the gyro to zero bias. However, it is not a real-time but an off-line compensation method.

There are two difficulties in compensating temperature error in the hemispherical resonator gyro. Firstly, the ambient temperature is unavailable. The interior of the hemispherical resonance gyro is a vacuum environment, and heat conduction and radiation are the only modes of heat exchange. The heat exchange modes make it difficult for the internal temperature of the gyro to change synchronously with the ambient temperature. So, it is impossible to use the external temperature environment to establish a temperature error model. Therefore, it is unreasonable to use the ambient temperature to establish a temperature error model. Secondly, the internal temperature cannot be measured. There is no temperature sensor in the hemispherical resonance gyro. If a temperature sensor is installed in the gyro, it will inevitably increase the design difficulty and space. Therefore, the internal temperature of the gyro cannot be measured directly. Hence, in this paper, to solve the above analyzed two problems, the availability and measurement of gyro internal temperature are analyzed, and A multiple regression based method for indirect compensation of HRG temperature error is proposed.

The content of the remaining paper is as follows. The relationship between internal temperature and resonant frequency is analyzed in “Analysis of the relationship between internal temperature and resonant frequency” section. “Model for indirect compensation of temperature error” section presents availability analysis of gyro internal temperature and multiple regression model. Then, to illustrate the effectiveness of temperature compensation model, a case study is conducted in “Experiments” section. Results are drawn in “Results” section, and this research is discussed in “Discussion” section.

## Methods

This section analyzes the relationship between internal temperature and resonant frequency firstly, and the one-to-one correspondence between them is obtained. Then, availability analysis of gyro internal temperature is presented. At last, a temperature error model for the hemispherical resonant gyro is established indirectly by transforming the independent variable of the model from the internal temperature to the resonant frequency.

### Analysis of the relationship between internal temperature and resonant frequency

#### Theoretical analysis

The hemispherical resonator is made of a fused quartz material. Its natural resonant frequency depends upon the material’s Young's modulus $E$, density $\rho$, and Poisson ratio $\mu$, as well as the thickness $h$ and radius $r$ of the harmonic oscillator hemispherical shell. When the temperature changes, the above parameters change accordingly, so that the resonant frequency also changes. Thus, the natural resonant frequency can be deformed to^8,9^:

1 $$f(T) = 1.5127\frac{h(T)}{{r^{2} (T)}}\left\{ {\left[ {\frac{E(T)}{{(1 + \mu (T))\rho (T)}}} \right]} \right\}^{\frac{1}{2}}$$

where $f(T)$ is the natural resonant frequency, $T$ is temperature.

According to Eq. (1), it may become extremely difficult to establish a theoretical model of resonant frequency and temperature. Therefore, an experimental method is used to establish a relationship between the gyro’s internal temperature and resonant frequency.

#### Experimental analysis

**Figure 1:**
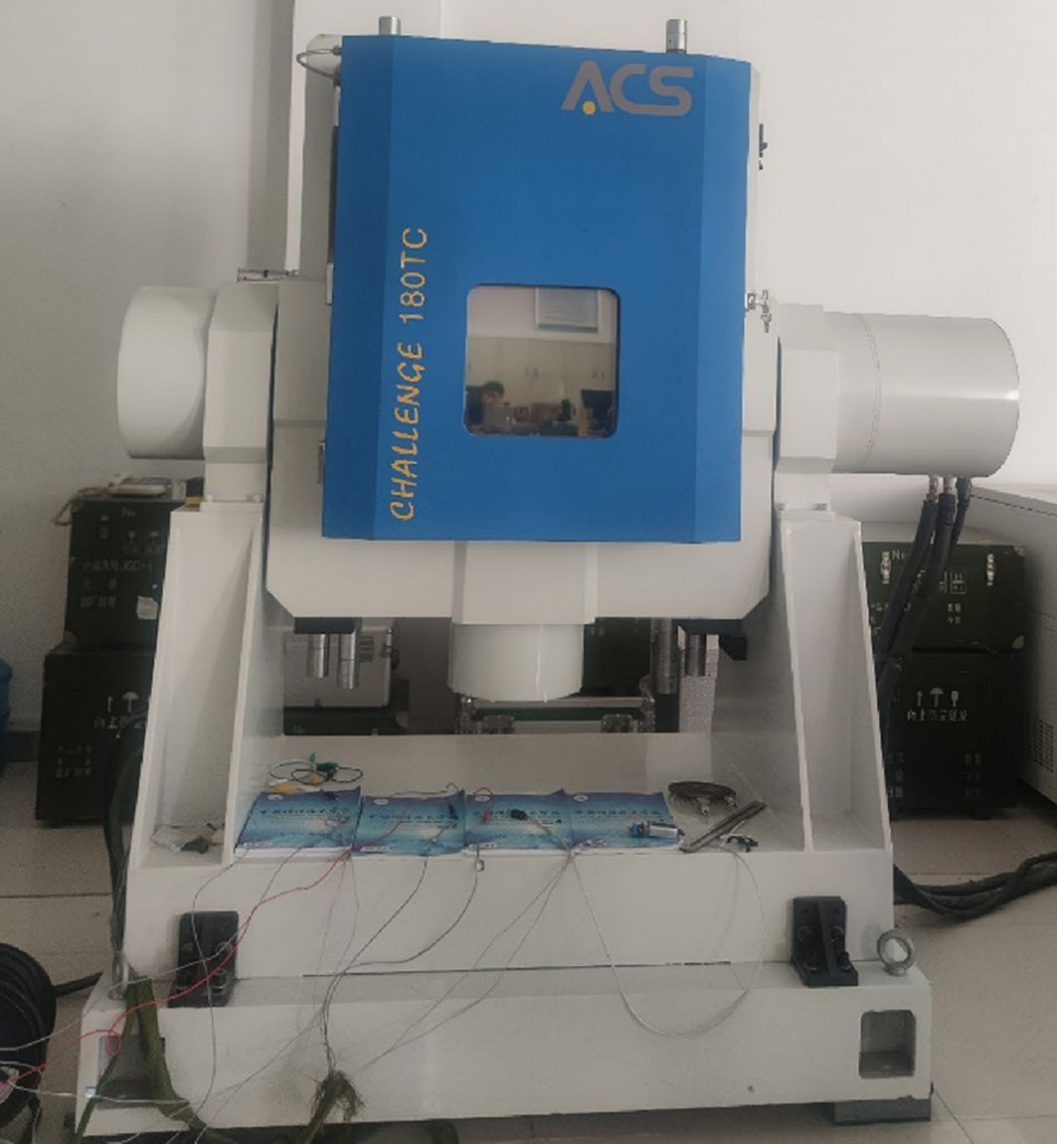
CH180TC temperature control box.

A constant temperature experiment of the hemispherical resonant gyro is designed to analyze the relationship between the resonant frequency and temperature. The required experimental equipment is as follows: KEITHLEY 2400 regulated power supply, CH180TC temperature control box, digital multimeter, Debugging industrial computer and 4005# hemispherical resonant gyro. The CH180TC temperature control box is shown in Fig. 1. Its technical specifications are as follows: temperature regulating range is greater than − 50 ~ 85 °C, temperature fluctuation is ≤  ± 0.5 °C, temperature deviation is ≤  ± 1 °C, temperature uniformity is ≤  ± 1 °C, linear temperature rise control is ≥ 5 °C/min, and average temperature drop control is ≥ 5 °C/min.

**Figure 2:**
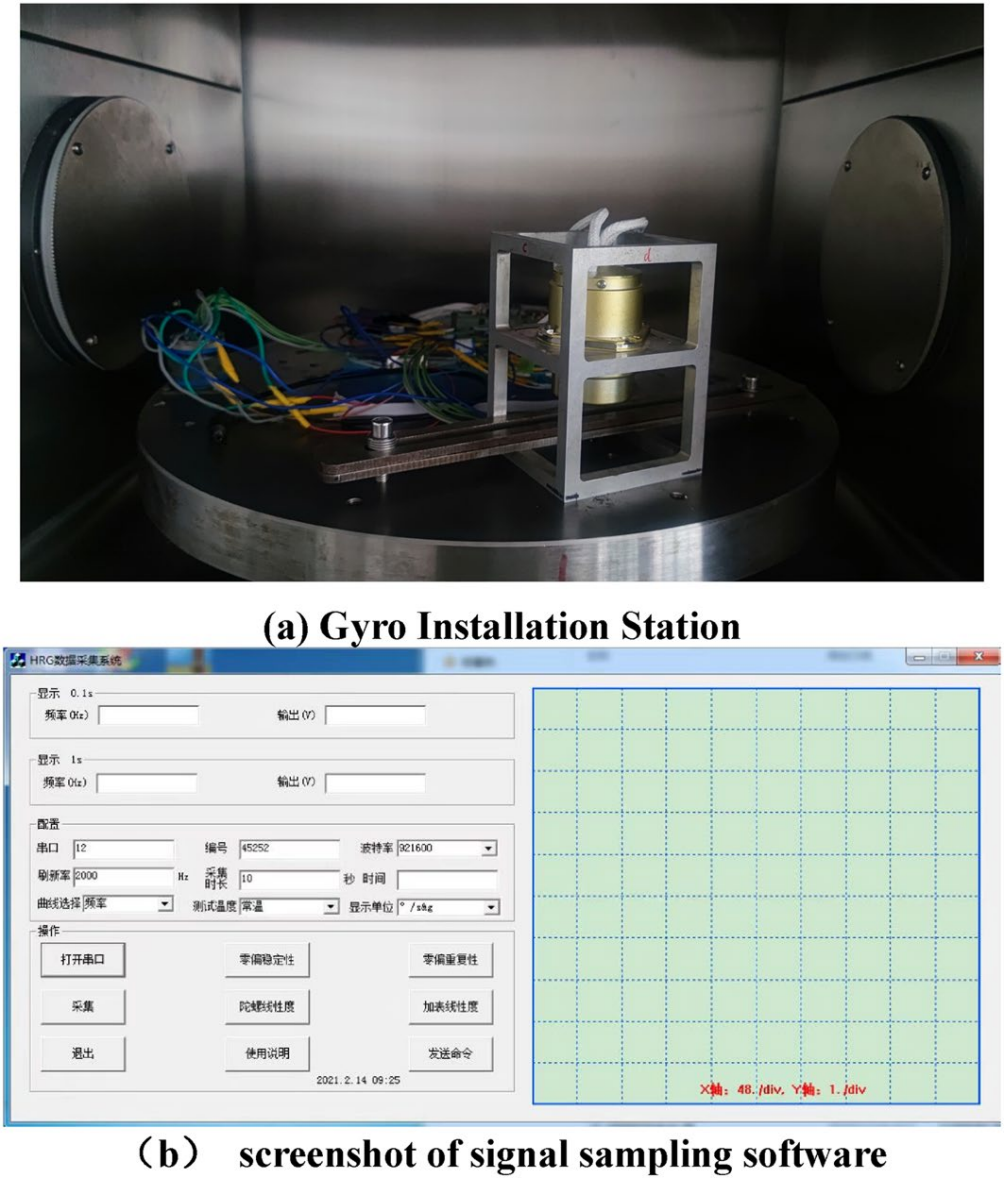
Photos of the experiment.

The experimental steps of the constant temperature experiment are as follows:

*Step 1* Fix the hemispherical resonance gyro in the temperature control box through the installation fixture. The input shaft of the gyro is installed in the sky direction, which is shown in Fig. 2a, and the screenshot of signal sampling software is shown in Fig. 2b. Keep the position of the gyro fixed throughout the test cycle;

*Step 2* Set the sampling interval and test time for the output of the gyro. After the temperature in the temperature control box reaches the expected value and becomes stable, maintain that temperature for 4 h to make the internal temperature of the gyro consistent with the box temperature. Then, the gyro starts up and data is collected. The data acquisition frequency is 1 Hz. After the gyro resonance frequency becomes stable, continue to collect data for 180 min;

*Step 3* Record the output voltage and resonant frequency of the gyro during the test.

**Figure 3:**
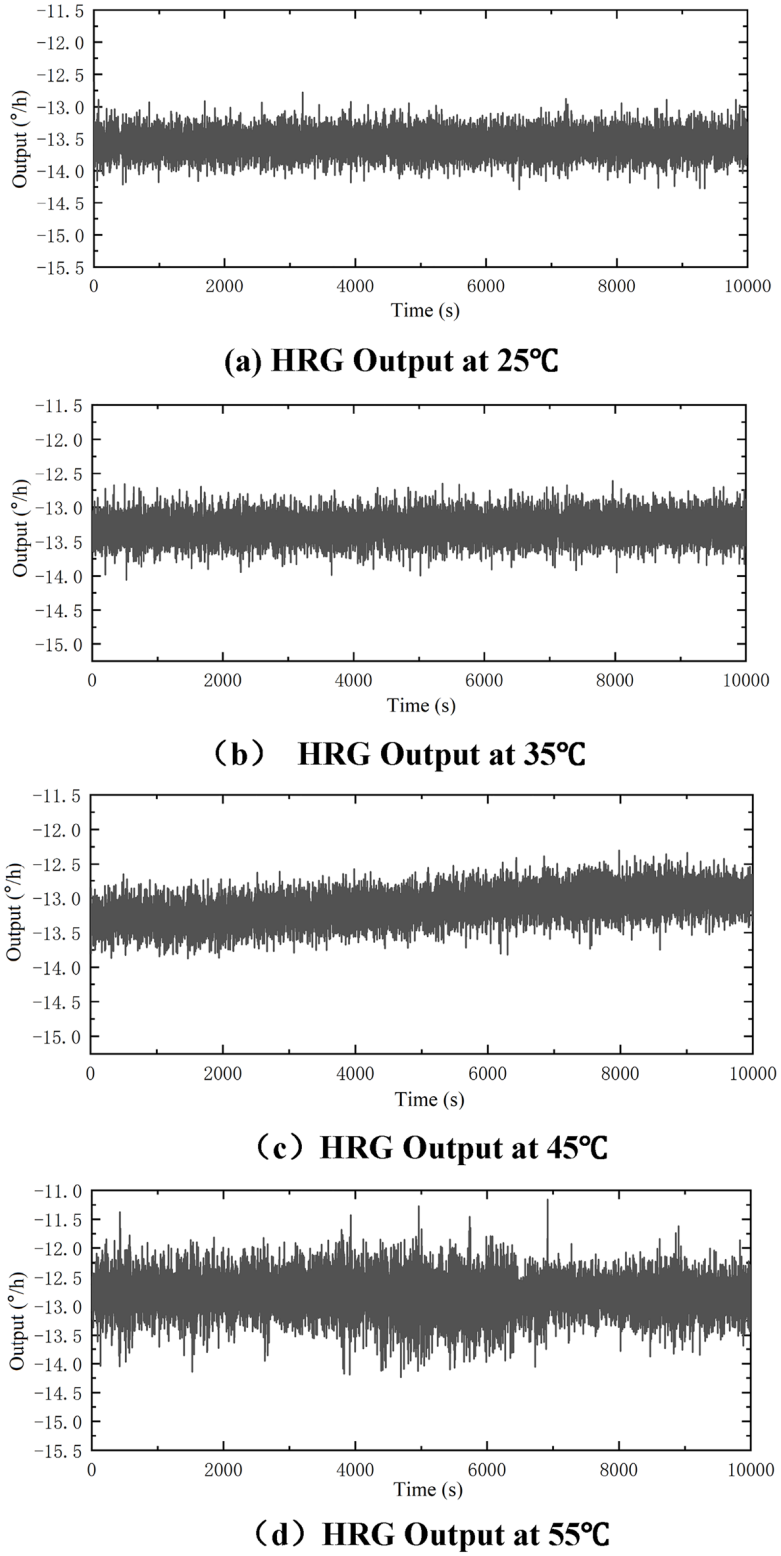
HRG output under constant temperature.

According to the need of the research and conditions of the laboratory, the maximum test temperature is set at 55 °C, the minimum temperature is set at 25 °C, making the whole temperature range of 30 °C. The temperature experiment is carried out at every 5 °C. Since the experimental dataset is extremely large, the original data only at 25 °C, 35 °C, 45 °C and 55 °C are selected as shown in Fig. 3.

The output parameters of the gyro at different temperatures are calculated, which can be reference values for temperature compensation effect at later stages. The results are shown in Table 1.

**Table 1:** Gyro output parameters under constant temperature.

Temperature (°C)	25	35	45	55
Output mean (°/h)	− 13.5751	− 13.296	− 13.0213	− 12.8032
Drift (°/h)	0.1924	0.1933	0.2530	0.3268

Next, the relationship between the resonant frequency and gyro internal temperature is established. In order to reduce the influence of random factors on the experimental results, each group of experiments was repeated three times. The average of the resonant frequency values of the three experiments is calculated as the resonant frequency at the corresponding temperature. The results are shown in Table 2.

**Table 2:** Gyro resonant frequency under constant temperature.

Temperature (°C)	Frequency of three experiments (Hz)			Frequency mean (Hz)
25	4960.341	4960.291	4960.291	4960.308
30	4962.628	4962.646	4962.646	4962.640
35	4964.921	4964.931	4964.942	4964.931
40	4967.230	4967.203	4967.236	4967.223
45	4969.469	4969.552	4969.652	4969.558
50	4971.950	4971.967	4972.011	4971.976
55	4974.492	4974.526	4974.538	4974.519

Reference^10^ shows that there is a linear relationship between the internal temperature and resonant frequency of the hemispherical resonant gyro. Therefore, based on the data presented in Table 2, the following linear relationship between the temperature and resonant frequency is established by the least square method:

2 $$f = 0.4726T + 4948.425$$

**Figure 4:**
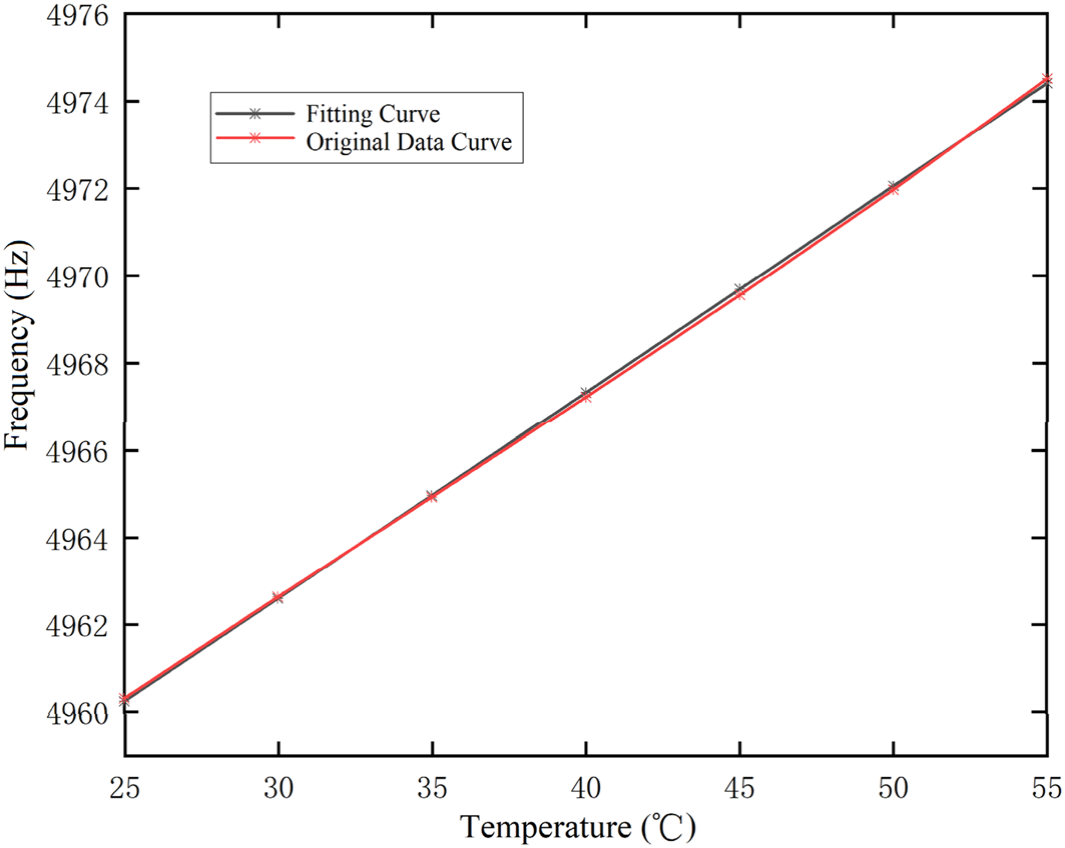
Fitting relationship between resonant frequency and temperature.

The comparison between the original data and the curve fitting data is shown in Fig. 4, where the temperature coefficient of the resonant frequency is 0.4726 Hz/(°C) and the linearity is better than 6 × 10^–6^.

The relative error in Eq. (2) for fitting the real frequency is shown in Table 3.

**Table 3:** The relative error in Eq. (2) for fitting the real frequency.

Temperature (°C)	Experimental frequency (Hz)	Fitting frequency (Hz)	Relative error
25	4960.308	4960.3240	− 1.37088E−05
30	4962.640	4962.603	− 7.45571E−06
35	4964.931	4964.966	7.04944E−06
40	4967.223	4967.329	2.13399E−05
45	4969.558	4969.692	2.69642E−05
50	4971.976	4972.055	1.58891E−05
55	4974.519	4974.418	− 2.03035E−05

The data presented in Table 3 depict two properties. Firstly, according to the linearity, there is a good linear functional relationship between the resonant frequency and internal temperature of the gyro. Secondly, according to the relative error in the curve fitting, the fitting accuracy of the linear function for the resonant frequency and temperature is very high.

### Model for indirect compensation of temperature error

#### Availability analysis of gyro internal temperature

As mentioned in “Introduction” sect, there are two difficulties in compensating temperature error in the hemispherical resonant gyro: the ambient temperature is unavailable and the internal temperature of the gyro is unmeasurable. The difficulty of indirect measurement of the gyro’s internal temperature has been resolved in “Model for indirect compensation of temperature error” section. However, the other difficulty is still there, *i.e.,* whether the internal temperature of the gyro is available to establish temperature error model? In order to resolve this problem, a temperature-rising experiment is designed.

**Figure 5:**
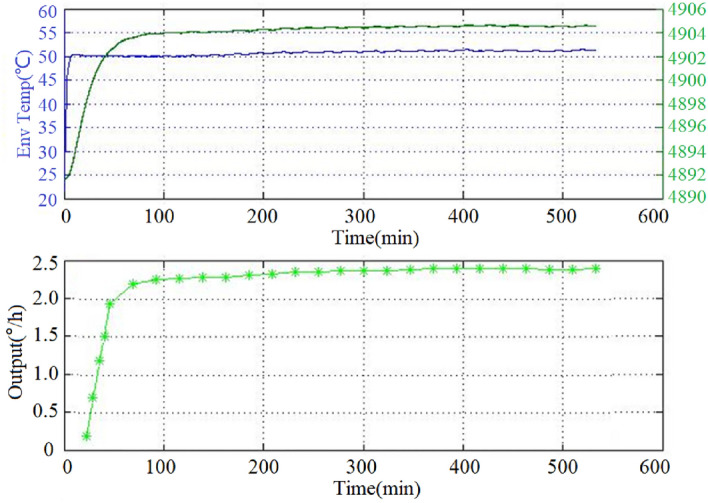
Test curve of ambient temperature, resonant frequency, and gyro output.

The initial temperature of the test chamber is the current room temperature of 24 °C, and the termination temperature is 55 °C. The input shaft of the gyroscope remains unchanged in a certain direction on a horizontal plane. The temperature control box starts to heat up upon the output of the gyro becomes stable after powering on the hemispherical resonant gyro. Then, the ambient temperature of the box, hemispherical resonator resonance frequency, and gyro output are collected. The data acquisition frequency is 1 Hz and the acquisition time is 540 min. The gyro output is filtered by the mean value, which is calculated for every 60 data points (i.e., 1 min). The obtained ambient temperature, resonant frequency, and gyro output curves are shown in Fig. 5. The "*" sign in the gyro output curve is the part of the gyro output value.

As can be seen in Fig. 5, the correlation between the gyro output and resonant frequency (representing the internal temperature) is significantly higher than that between the gyro output and ambient temperature.

The covariance difference $\rho$ between the two sequences can characterize the correlation between them. When $\rho > 0$, the correlation of the two sequences is positive; when $\rho < 0$, the correlation is negative. Further, the greater the absolute value of $\rho$, the stronger is the correlation of the two sequences. The covariance between the gyro output and resonant frequency is found to be 2.485, and that between the gyro output and ambient temperature is 0.764. The above data indicates that the gyro output has a certain positive correlation with both resonant frequency and ambient temperature, but the correlation between the gyro output and resonant frequency is much higher than that between the gyro output and ambient temperature. Besides, Reference^11^ points out that the influence of temperature on gyro output is independent and regular. Therefore, the temperature error model of the gyro can be established based on the resonant frequency (i.e., gyro internal temperature).

#### Multiple regression model

The correlation between the gyro output and the gyro internal temperature (i.e., resonant frequency) is analyzed in “Design of temperature-rising experiment and clarification of compensation effect” section, and the conclusion is drawn that the temperature error model can be established based on the gyro internal temperature (i.e., resonant frequency). Therefore, the temperature error model of the hemispherical resonant gyro is constructed with the resonant frequency as the independent variable.

When the hemispherical resonator gyro works, the change in thermal field and the uncertainty in heat conduction lead to a difference in the resonator resonant frequency and its rate of change with time. Therefore, the temperature error model of the hemispherical resonator gyro is established based on the frequency and its rate of change.

Common fitting models include neural network model, polynomial model, and multiple regression model. The neural network model has the self-learning ability, but it needs a large number of training samples, and its fitting effect is related to the quality of the parameter training. The principle of polynomial model is simple, but there are some problems, such as few independent variables and relatively low fitting accuracy. The multiple regression model has the advantages of simple principle, small amount of calculation, sufficient independent variables, and high fitting accuracy. In this section, the multiple regression model is used to establish the temperature error model of the gyro. The multiple regression model with indefinite order can be expressed as follows^12^:

3 $$\tilde{\omega }(f) = \omega_{0} + a_{1} f{ + }a_{2} f^{\prime} + a_{3} (f^{\prime})^{2} + a_{4} (ff^{\prime}) + a_{5} f^{2} + \cdots$$

where $\tilde{\omega }(f)$ is the compensation value of temperature error, $\, \omega_{0}$ is the zero order term, and $a_{1} {{\sim }}a_{5}$ are coefficients.

The order of the multiple regression model can be determined according to the following principles^13^:

1. If the gyro drift can meet the gyro performance requirements after compensation of the established *n*-order model, then the accuracy of the model will meet the requirements.
2. If the accuracy of the established (*n* + 1) -order model is similar to that of the *n*-order model, then the order of the model will not increase.
3. If the (*n* + 1)-order coefficient of the established (*n* + 1)-order model is close to or equal to 0, then the order of the model will not increase.

## Experiments

### Design of temperature-rising experiment and clarification of compensation effect

#### Design of temperature-rising experiment and original data

The steps of the temperature-rising experiment are as follows:

1. Fix the hemispherical resonance gyro in the temperature control box through the installation fixture. Install the gyro input shaft in the sky direction (same as in Fig. 2), and keep the gyro position fixed throughout the test cycle.
2. Heating process experiment. Control the temperature in the box at 25 °C, and keep the gyro warm for 2 h after startup. Open the serial port to collect gyro data and control the temperature rise in the box at the same time. The heating rate in the box is kept ≥ 5 °C/min, so that the temperature in the box can be quickly increased to 55 °C within 6 min. Collect the output voltage and resonant frequency of the hemispherical resonant gyro. The sampling frequency is set to 1 Hz and the acquisition time is 3 h.

**Figure 6:**
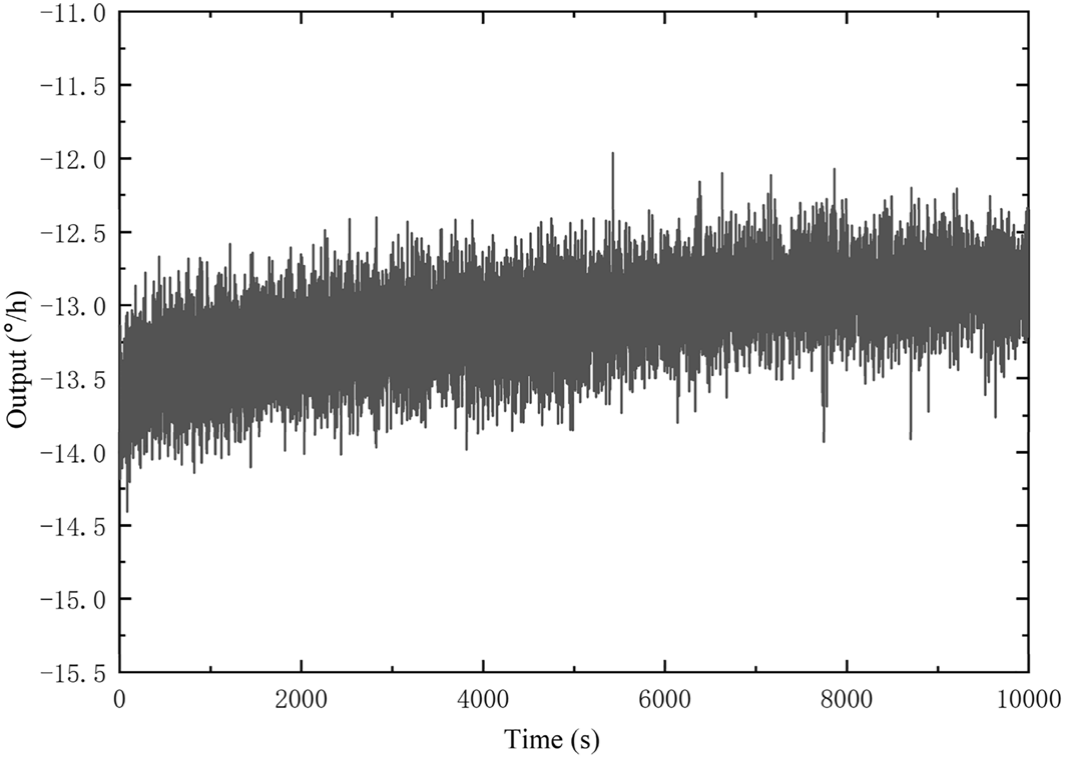
Gyro output data.

**Figure 7:**
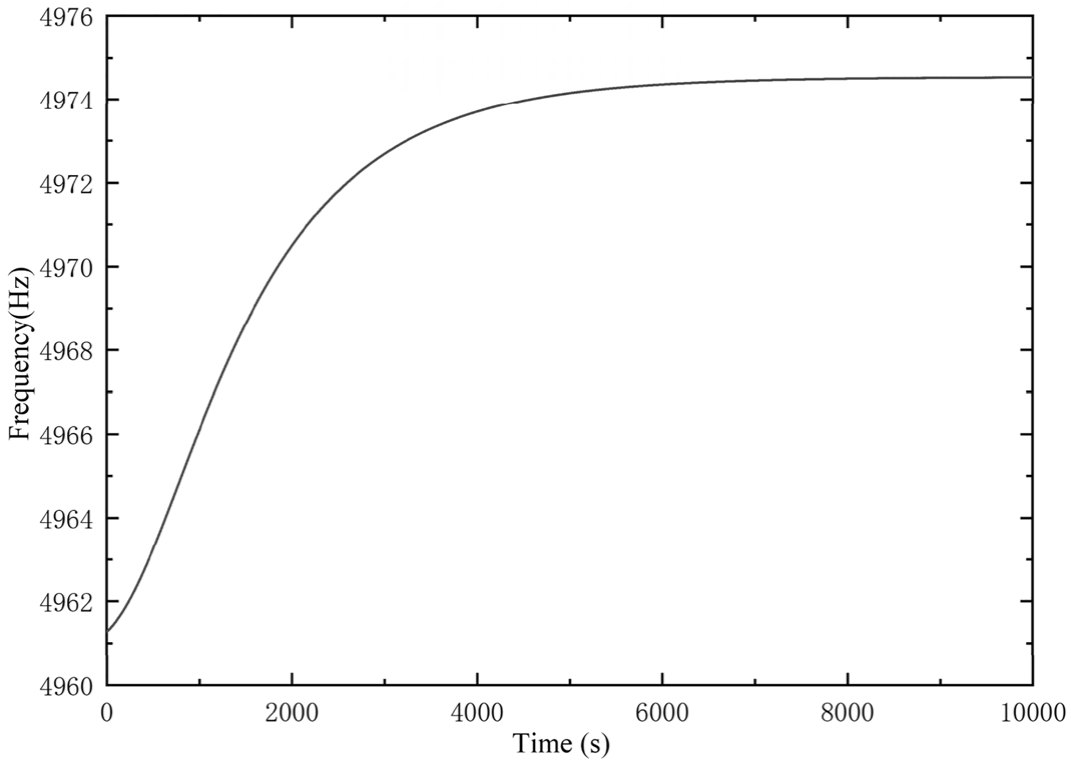
Gyro resonant frequency.

According to the above experimental method, a set of the original output data of the gyro is shown in Fig. 6, and their resonance frequency is shown in Fig. 7.

#### Model determination and verification of compensation effect

According to the principle of determining the order of a multiple regression model, the model for compensating temperature error is obtained as follows:

4 $$\tilde{\omega }(f) = \omega_{0} + a_{1} f{ + }a_{2} f^{\prime} + a_{3} (f^{\prime})^{2} + a_{4} (ff^{\prime})$$

where $\omega_{0} = - 534 {\text{.571849}}$°/h, $a_{1} = 0 {\text{.104859}}$°/h/Hz, $a_{2} = 75640 {\text{.492800}}$°/h/Hz, $a_{3} = 2620 {\text{.369671}}$°/h/Hz, and $a_{4} = - 15 {\text{.218514}}$°/h/Hz.

The compensation effect of the error model is verified from two aspects: one is to compare the gyro drifts before and after the compensation, and the other is to test the stability of the gyro output before and after the compensation.

**Figure 8:**
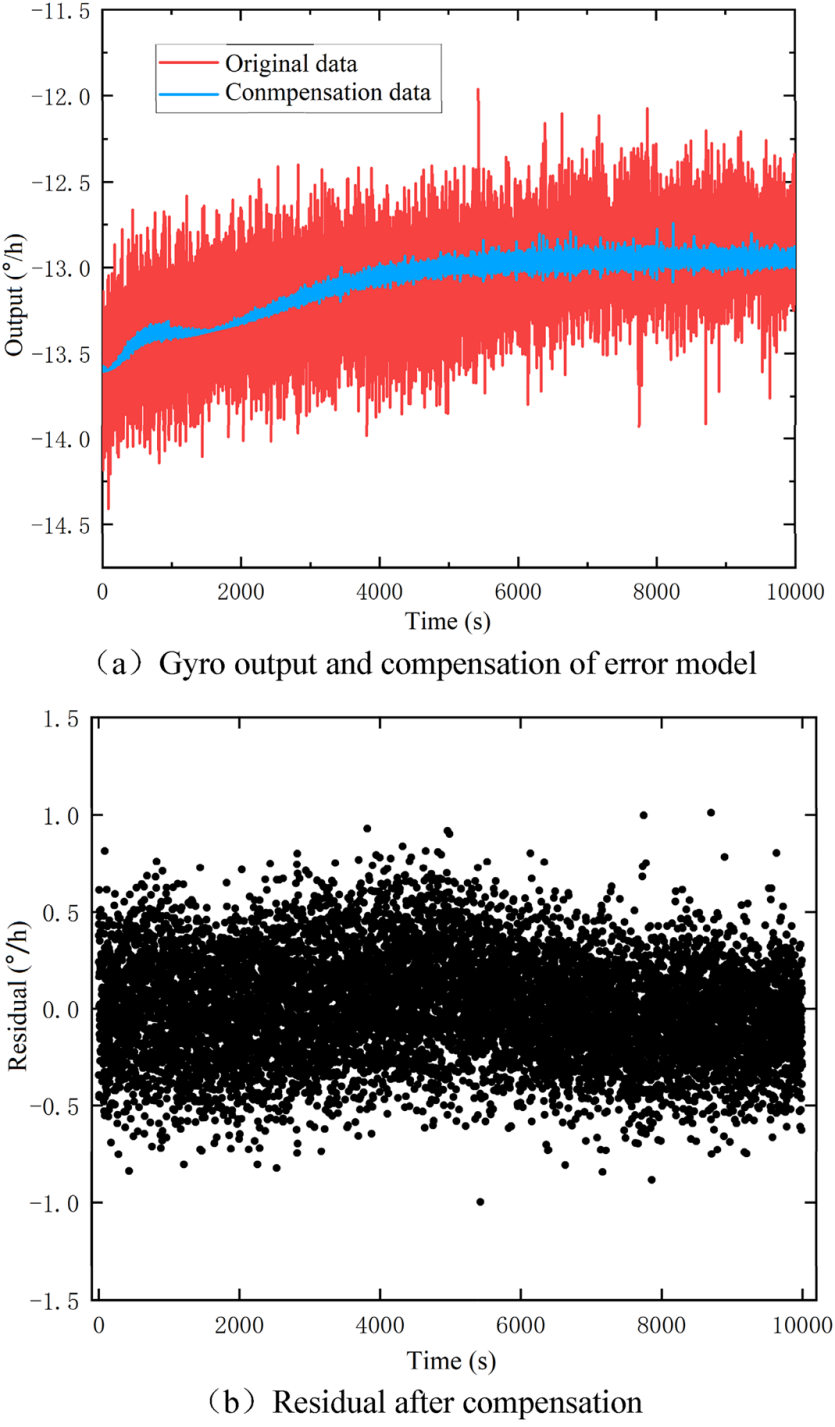
Compensation effect of temperature-rising experiment.

The compensation of the temperature error model to the original output data of the gyro is shown in Fig. 8.

Comparing Fig. 8a,b, it can be seen that the trend term contained in the residual diagram is significantly reduced. The residual sequence is basically close to the zero mean sequence. The gyro drift before the compensation of the temperature error is $\sigma = 0 {\text{.328894}}$°/h. After the compensation of temperature error, the gyro drift becomes $\sigma = 0 {\text{.202069}}$°/h. Referring to the gyro random drift at a constant temperature in Table 1, the following conclusions can be drawn: (1) After the compensation, the gyro drift value decreases by 62.76%, indicating that the compensation of the temperature error is effective; (2) The gyro drift after the compensation is close to the gyro random drift under a constant temperature, which indicates that the temperature error model effectively eliminates the influence of temperature change on the measuring accuracy of the gyro, so that the accuracy under variable temperature is equivalent to that under a constant temperature.

In order to further verify the compensation effect of the error model, the zero mean test and stability test are carried out on the compensated output series. The mean value of the output series after compensation is found to be 1.00251e-06°/h, which meets the requirements of the zero mean index of the gyro after the compensation. The ADF (August Dickey Fuller) method^14^ is used to test the stability of the gyro output series. The stability is tested through three models, and the test order is model 3, model 2 and model 1^15^:

5 $${\text{Model}}\;1:\Delta \omega_{t} = \delta \omega_{t - 1} + \sum\limits_{i = 1}^{m} {\beta_{i} \Delta \omega_{t - i} } + \varepsilon_{t}$$

6 $${\text{Model}}\;2:\Delta \omega_{t} = \alpha { + }\delta \omega_{t - 1} + \sum\limits_{i = 1}^{m} {\beta_{i} \Delta \omega_{t - i} } + \varepsilon_{t}$$

7 $${\text{Model}}\;3:\Delta \omega_{t} = \alpha { + }\beta t{ + }\delta \omega_{t - 1} + \sum\limits_{i = 1}^{m} {\beta_{i} \Delta \omega_{t - i} } + \varepsilon_{t}$$

where $\Delta \omega_{t} = \omega_{t} \, - \omega_{t - 1}$, $\alpha$ is a constant item, $\beta$ is a trend item, $\varepsilon_{t}$ is residual, and $m$ is the auto-regressive order.

In the ADF test, the original hypothesis is H0: $\delta = 0$, i.e., the output sequence has unit root and the sequence is not stationary. Alternative hypothesis is H1: $\delta \ne 0$, i.e., the output sequence has no unit root and the sequence is stationary. The program package adf.test() of MATLAB is used to test the stability of the sequence. The obtained result show that the output series is not stable before compensation, and it becomes stable only after the compensation, which indicates that the compensation of temperature error is effective.

### Design of temperature-dropping experiment and clarification of compensation effect

#### Design of temperature-dropping experiment

The steps of the temperature-dropping experiment are as follows:

1. Fix the hemispherical resonance gyro in the temperature control box through the installation fixture. Install the gyro input shaft in the sky direction (same as in Fig. 2), and keep the gyro position fixed throughout the test cycle.
2. Cooling process experiment. Control the temperature in the box at 55 °C, and keep the gyro warm for 2 h after startup. Open the serial port to collect the gyro data and control the temperature drop in the box at the same time. The cooling rate in the box is ≥ 5 °C/min, so that the temperature in the box can be quickly dropped to 25 °C within 6 min. Collect the output voltage and resonant frequency of the hemispherical resonant gyro. The sampling frequency is set to 1 Hz and the acquisition time is 3 h.

#### Verification of compensation effect

Equation (4) is applied to compensate the temperature error in the cooling experiment. The compensation effect of the error model is verified from two aspects: one is to compare the gyro drifts before and after compensation, and the other is to test the stability of the gyro output before and after compensation.

**Figure 9:**
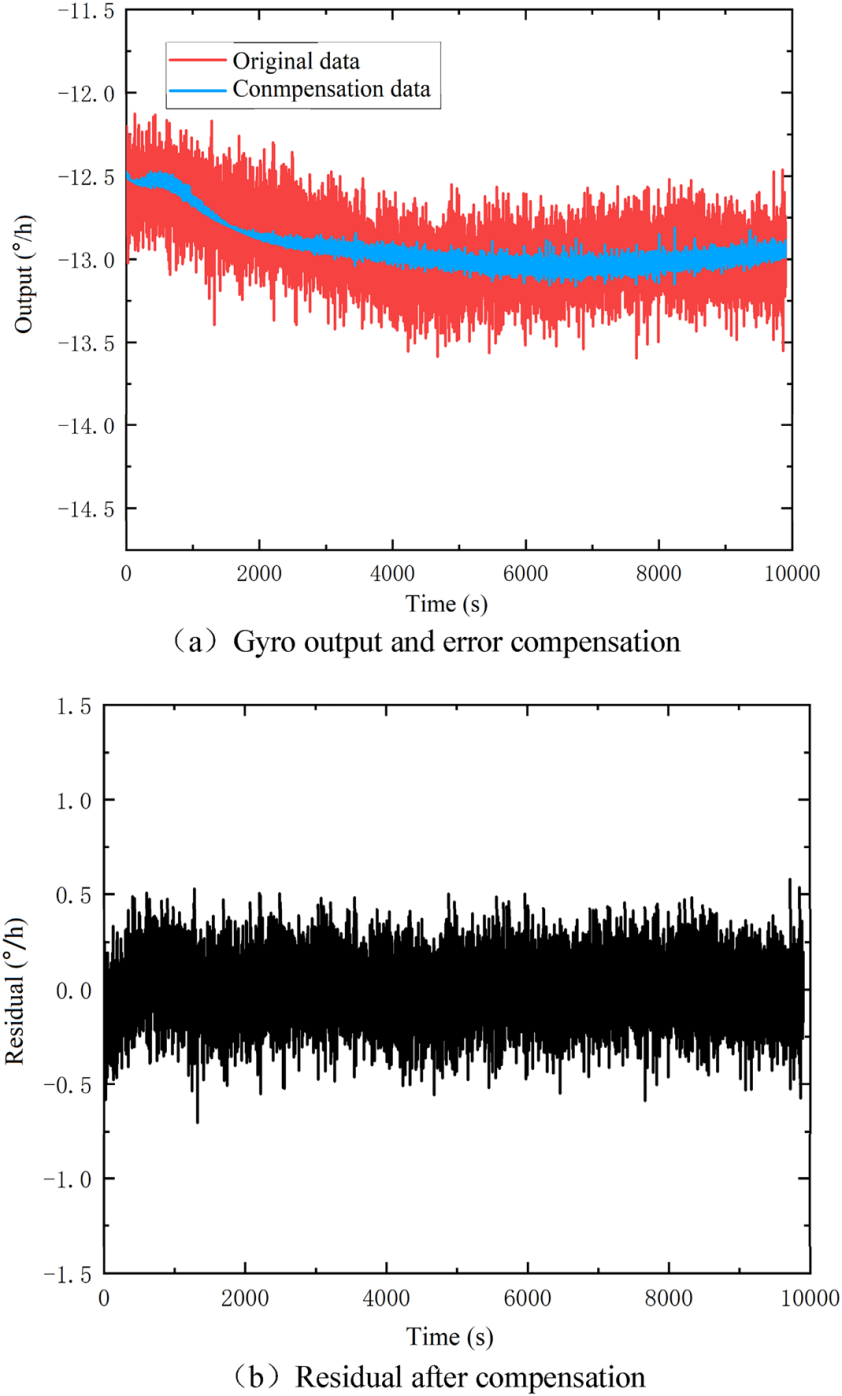
Compensation effect of temperature-dropping experiment.

In the cooling experiment, the compensation effect of the temperature error model for the gyro output is shown in Fig. 9.

Comparing Fig. 9a,b, it can be seen that the trend term contained in the residual diagram is significantly reduced. The residual sequence is basically close to the zero mean sequence. The gyro drift before the compensation of temperature error is found to be $\sigma = 0 {\text{.309764}}$°/h. After the compensation of temperature error, the gyro drift is found to be $\sigma = 0 {\text{.208621}}$°/h. Referring to the gyro random drift at a constant temperature as reported in Table 1, the following conclusions can be drawn: (1) After compensation, the gyro drift value decreases by 48.48%, indicating that the compensation of temperature error is effective; (2) The gyro drift after the compensation is close to the gyro random drift under a constant temperature. It depicts that the temperature error model effectively eliminates the influence of temperature change on the measuring accuracy of the gyro, so that the accuracy under variable temperature is equivalent to that under a constant temperature.

In order to further verify the compensation effect of the error model, the zero mean test and stability test are carried out on the compensated output series. The mean value of the output series after compensation is found to be -1.80202e-04°/h, which meets the requirements of the gyro zero mean index after compensation. The ADF (August Dickey Fuller) method is used to test the stability of the gyro output series. The obtained results show that the output series is not stable before compensation, and it becomes stable only after compensation. It indicates that the compensation of temperature error is effective.

### Comparison of compensation effect

The bias stability is the dispersion degree of gyro output around its mean value (i.e., bias), which is an important index to evaluate the gyro measurement accuracy. Therefore, the bias stability is taken as the basis for comparing the compensation effects of different methods.

**Figure 10:**
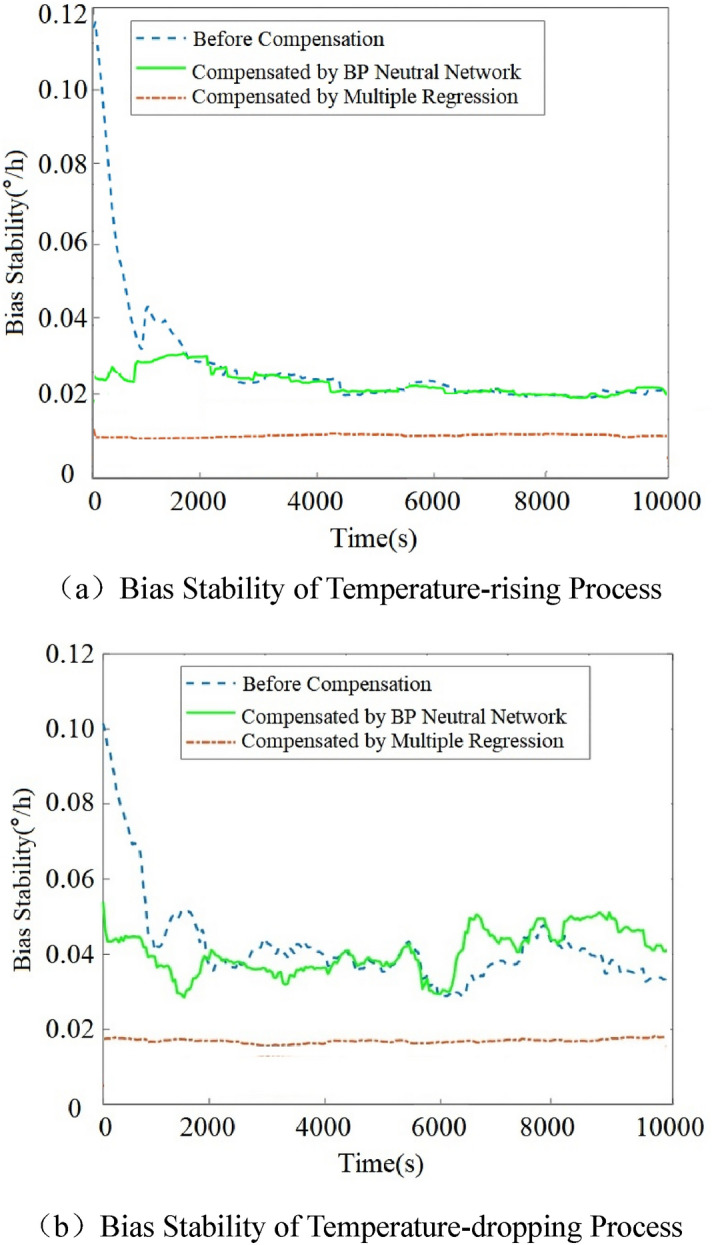
Bias stability of HRG before and after compensation.

For the temperature-rising and temperature-dropping experiments of the HRG, the compensation effect of the multiple regression model is compared with the BP neural network^4^. The bias stability of the two models before and after compensation is shown in Fig. 10.

It can be seen from Fig. 10 that the gyro bias stability after compensation by multiple regression model is less than that by BP neural network, indicating that the gyro measurement accuracy after compensation by multiple regression model is higher. This is because the independent variable of the neural network model is the resonant frequency, while the independent variable of the multiple regression model includes the resonant frequency, the frequency change rate and the coupling term, which can more deeply fit the corresponding relationship between gyro drift and temperature.

Based on the compensation results of the temperature-rising and temperature-dropping experiments, it can be observed that: (1) the model for compensating temperature error in the same hemispherical resonator gyro is universal in the temperature-dropping and temperature-rising experiments. Then, in engineering applications, the temperature error model for the same gyro can realize the real-time compensation of temperature error. (2) The indirect compensation method based on the multiple regression model can effectively eliminate the influence of temperature, and make the measuring accuracy of the gyro under variable temperature consistent with that under a normal temperature. (3) The temperature drift compensation accuracy of multiple regression model is higher than that of BP neural network mode.

## Results

The compensation of temperature error in the hemispherical resonator gyro under variable temperature is studied. The problems of compensating temperature error in the hemispherical resonator gyro are solved, such as "the external temperature is unavailable and the internal temperature is unmeasurable". The relationship between the internal temperature and resonant frequency is analyzed both theoretically and experimentally, and a mathematical model of resonant frequency and internal temperature is established. The correlation between the gyro output and the resonant frequency is studied, and it is concluded that the resonant frequency can be used for modeling temperature error. Finally, the temperature error model of the hemispherical resonator gyro is established based on a multiple regression theory. After verification, the following conclusions are drawn:

1. Under the condition of temperature rising, the gyro drift after compensation decreases by 62.76%, and becomes similar with the gyro random drift under a constant temperature, indicating that the measuring accuracy of the gyro under variable temperature is equivalent to that under a constant temperature after compensation;
2. Under the condition of temperature-dropping, the gyro drift after compensation decreases by 48.48%, and becomes similar to the gyro random drift under a constant temperature, indicating that the measuring accuracy of the gyro under variable temperature is equivalent to that under a constant temperature after compensation;
3. Temperature compensation method of HRG in this paper realizes real-time compensation, that is because: First, the temperature compensation model takes the resonant frequency and frequency change rate as independent variables, and the resonant frequency can be measured in real time; Second, for the same gyro, the same compensation model has a good compensation effect both in the temperature-rising and temperature-dropping process; Third, the temperature compensation model is an explicit model, and the corresponding gyro output compensation value can be calculated for each resonant frequency. So the method in this paper can realize real-time compensation.

## Discussion

This research can improve the measurement accuracy of hemispherical resonator gyro under variable temperature conditions, and then promote the practical application of hemispherical resonator gyro in satellites and spacecraft. However, this study also has its limitations. The experiment is carried out in the range of 25–55 °C, and the applicability of the error model outside this temperature range is unknown.

## Data Availability

The datasets generated and analyzed during the current study are not publicly available because technologies related to hemispherical resonant gyro are confidential, but they are available from the corresponding author on reasonable request.

## Abbreviation

HRG: Hemispherical resonant gyro
